# L’hôtel-Dieu of Paris

**Published:** 1859-03

**Authors:** 


L'HÔTEL-DIEU OF PARIS.—This ancient hospital is now being demolished, to make room for a new institution in a more healthy location, and for the purpose of providing better sewerage and ventilation, which were the faults of the old building. The Hôtel-Dieu had nearly one thousand beds, and received annually about twelve thousand patients, who were nursed by forty Sisters of Charity, mostly Roman Catholics. The founding of this hospital is said to date back as far as the year 660; but this point is disputed. We extract the following paragraph from the *Medical Times and Gazette*, which will prove interesting to our readers:—

"It is generally believed, but without proof, that the foundation of the Hôtel-Dieu was due to St. Landry, Bishop of Paris, in the seventh century. The canons of Nôtre Dame only possessed at first the half of this establishment; the other part was ceded to them in 1202, by Renaud, Bishop of Paris. At that time not only the poor sick, but also the healthy poor, were admitted into it; it was a hospital in the true sense of the word. Phillippe Auguste was the first king that gave donations to it. We read in one of his letters:—'We gave to the Maison de Dieu de Paris, for the poor there, all the straw in our room and house in Paris, each time that we leave the town to sleep elsewhere.' The increase of population brought an increase of sick, and thus the Hôtel-Dieu became insufficient for their accommodation. In 1217 Dean Stephen, conjointly with the Chapter, charged four priests and four clerks with the spiritual care of it; thirty priests and twenty-five clerks provided for the wants of the sick. Under St. Louis, the hospital was rebuilt and enlarged. It then took the name of Hôtel Nôtre Dame, and was exempt from all taxes. In 1511, the Rue des Sablons was closed, in order to increase its size. In 1531, Cardinal Duprat built a ward, which was called before the Revolution, Salle du Legat. In 1602, Henry IV. constructed the Salle St. Thomas; in 1606, the Salle St. Charles was finished, through the liberality of Pomponne de Bellièvre. Louis XIV., like his predecessors, favored the hospital. In 1772, the accumulation of sick was so great, that as many as eight patients were put in one bed; and on the morrow, almost always three or four were found dead. The mortality rapidly increased, and the Hôtel-Dieu became a permanent source of infection to the city; and this brought about an improvement in its administration. Under the Revolution, the Hôtel-Dieu was rebaptized, and called Maison de la Humanité. The Hôtel-Dieu is now about to be entirely demolished."

